# Pickering Emulsion Stabilized by *Pueraria lobata*-Based Stabilizer: From a Passive Carrier to an Active Partner for Oral Capsaicin Delivery

**DOI:** 10.3390/foods15152617

**Published:** 2026-07-26

**Authors:** Qiongliu Yu, Yikang Ding, Hanyu Wu, Min Luo, Qunying Zhang, Guiming Yan, Ye Yang

**Affiliations:** 1 School of Pharmacy, Anhui University of Chinese Medicine, Hefei 230012, Chinaqunyingzhang701025@ahtcm.edu.cn (Q.Z.); 2 School of Nursing, Anhui University of Chinese Medicine, Hefei 230012, China; wuweizi@ahtcm.edu.cn; 3 Anhui Provincial Key Laboratory of Research & Development of Chinese Medicine, Hefei 230012, China; 4 Anhui Provincial Key Laboratory of Pharmaceutical Preparation Technology and Application, Hefei 230012, China

**Keywords:** capsaicin, *Pueraria lobata*, microwave-assisted enzymolysis, Pickering emulsion, gastrointestinal irritation

## Abstract

Capsaicin is a bioactive substance with diverse health-promoting properties, but its intense pungency and irritant side effects limit oral application. This study developed a *Pueraria lobata* particle-based Pickering emulsion (PE) as an oral delivery platform for capsaicin. Modified *Pueraria lobata* particles (MP-ps) were prepared using a microwave-assisted enzymolysis technique (15% hydration, 252 J/g microwave energy and 8 h pullulanase hydrolysis). MP-ps exhibited surface cracks and pores, altered starch crystallinity, and enhanced water-holding capacity and swelling power. Compared with original *Pueraria lobata* particles, MP-ps avoided the burst release of puerarin in the upper gastrointestinal tract, dropping from approximately 54% to 20%. An optimized capsaicin-loading PE with excellent physical stability was obtained from 4% MP-ps and a 9:1 (*v*/*v*) water-to-corn oil ratio, exhibiting complete core–shell architecture and oil-phase sequestration of capsaicin in TEM images. Gastrointestinal transit analysis in male KM mice indicated that MP-p-based PE reduced the exposure of the encapsulated lipophilic substance in the stomach and small intestine and promoted its accumulation in the colon. Irritation assays in male KM mice and Sprague-Dawley rats further demonstrated that this effective encapsulation alleviated the gastrointestinal irritation of capsaicin and improved its palatability. This study provided a food-derived material and an easy-to-build PE platform for oral lipophilic/irritant substance delivery, with potential for future health-regulating applications.

## 1. Introduction

Capsaicin (Cap), the major active ingredient in chili pepper, exhibits various physiological benefits such as anti-inflammatory [1], antioxidant [2], anticancer [3], anti-obesity [4], and abirritation [5] characteristics. As a functional food ingredient, it can also modulate the gut microbiota and inhibit pathogenic colonization [6]. However, the application of capsaicin in health-regulating remains limited by intense spiciness, irritation, and lipophilicity [7]. Therefore, developing food-derived delivery systems that can reduce capsaicin-related irritation while improving its gastrointestinal delivery performance is of practical importance.

In recent years, Pickering emulsion (PE) has emerged as a novel delivery platform for encapsulation and protection of bioactive ingredients [8]. PE has the unique advantage of high stability, because it employs solid particles as the stabilizer to create a robust barrier at between-phase interfaces. Since consumer preferences are increasingly shifting towards natural and healthy ingredients, Pickering stabilizers of natural origin [9], such as proteins [10], polysaccharides [11] and lipid crystals [12], are gaining significant popularity in PE construction. Among these materials, starch-based particles are particularly attractive because starch is one of the common polysaccharides in nature and is widely present in plants. Starch granules obtained from various food sources, such as potato [13], cassava [14], and corn [15], have been successfully applied to the construction of PEs. Physical [16], chemical [17], and enzymatic modifications [18] can further tailor the surface wettability of starch granules precisely. In addition, food-derived particle stabilizers may offer additional functional attributes due to their inherent bioactivities. However, the direct use of starch-rich food-derived plant materials with inherent bioactive components remains insufficiently explored.

The successful PE constructions based on starch granules might lay the groundwork for the use of starch-rich materials as Pickering stabilizers. The dried root of *Pueraria lobata* (Willd.) Ohwi (*P. lobata*) is a traditional food and medicinal material with a long history of use [19]. Given the inherent high starch content in the root of *P. lobata*, up to 53% to 68%, it has the potential to act as a solid particle stabilizer for PE construction through altering the surface wettability. Meanwhile, it can contribute multiple bioactive compounds. The PE system constructed from this natural-derived material of *P. lobata* not only enables the efficient delivery of lipophilic/irritant substances like capsaicin, but also leverages inherent active components, including puerarin, dietary fiber and resistant starch (RS), to create beneficial effects as well [20].

To overcome the challenges of irritation and lipophilicity in capsaicin application, this study was conducted to develop and evaluate an oral PE system, using *P. lobata* root-derived particles as Pickering stabilizers. A green and efficient technique of microwave-assisted enzymolysis was used, and the structure, morphology and surface wettability of modified *P. lobata* particles were characterized. After optimizing the parameters of stabilizer preparation, stabilizer amount, and oil-to-water ratio, capsaicin-loaded PEs were constructed and subjected to gastrointestinal transit analysis, animal preference testing, and gastrointestinal mucosal irritation evaluation. This study provided a simple, green, and low-cost framework for oral lipophilic/irritant substance delivery systems, which should be universal in development of health-regulating supplements.

## 2. Materials and Methods

### 2.1. Materials

The root of *P. lobata* was purchased from Anhui Kangmei Pharmaceutical Co., Ltd. (Bozhou, China). Pullulanase (100 U/g) was purchased from Shanghai Macklin Biochemical Technology Co., Ltd. (Shanghai, China). Corn oil, used as the oil phase of PE, was purchased from Lu Hua Group (Yantai, China). Puerarin (purity ≥ 97%), capsaicin (Cap, purity 99%), porcine pancreatin, and porcine pepsinum were obtained from Yuanye Biological Co., Ltd. (Shanghai, China). Nile Red, hematoxylin and eosin were purchased from Beijing Solarbio Technology Co., Ltd. (Beijing, China). All the other chemicals used in the study were of analytical grade and were obtained commercially.

Specific-pathogen-free Kunming (KM) mice (male, 6 weeks old, 22 ± 3 g) and Sprague-Dawley rats (male, 8 weeks old, 220–240 g) were purchased from the Animal Center of the Anhui University of Chinese Medicine (License: SCXK(Liao) 2020-0001). Animals were fed a standard diet under controlled conditions (12/12 h light/dark cycle, 45–50% humidity, and 20–22 °C) and were acclimated for 7 days prior to any experiment. All experimental animals were euthanized by CO_2_ inhalation in accordance with the approved animal study protocol. The animal study protocol was approved by the Animal Ethics Committee of Anhui University of Chinese Medicine (protocol code AHUCM-mouse-2023052).

### 2.2. Preparation of Modified P. lobata Particles

The root of *P. lobata* was pulverized and passed through a 100-mesh sieve to obtain original *P. lobata* particles (OP-ps). The modification of OP-ps was achieved using microwave-assisted enzymolysis technology with optimized parameters, including hydration degrees, microwave energy, and pullulanase hydrolysis. Microwave treatment was performed using a microcomputer microwave chemical reactor (WBFY-201, Shanghai Muzo Scientific Instruments Co., Ltd., Shanghai, China). Modified *P. lobata* particles (MP-ps) were obtained from low (10%, L)- or high (15%, H)-hydration-degree and low (126 J/g, L)- or high (252 J/g, H)-energy-density microwave processing of *P. lobata* particles followed with 8 h pullulanase hydrolysis [21], and were respectively designated as MP_L_-p_L_, MP_L_-p_H_, MP_H_-p_L_, and MP_H_-p_H_.

### 2.3. Characterization of P. lobata Particles as a Pickering Stabilizers

The structure of *P. lobata* particles was characterized using scanning electron microscopy (SEM; SU-8100, Hitachi, Tokyo, Japan) for morphology, polarizing microscopy (PLM; Axio Scope.A1, Zeiss, Oberkochen, Germany) for crystalline features, and laser diffraction (Bettersizer 2600E, Bettersize Instruments Ltd., Dandong, China) for particle size distribution, from which the D_10_, D_50_, and D_90_ values (representing the particle diameters at 10%, 50%, and 90% cumulative volume, respectively) were obtained. The surface wettability of *P. lobata* particles was evaluated indirectly by measuring their water-holding capacity (WHC), solubility (S), and swelling power (SP). WHC was determined by hydrating samples (1.00 ± 0.01 g) in 20 mL of deionized water under vortex agitation (30 min, 25 °C) and centrifuging them (5000× *g*, 10 min), with gravimetric measurement of the decanted hydrates [22]. Solubility and swelling power were determined using a modified gravimetric method [23]. Briefly, a 1% (*w*/*w*) aqueous dispersion of OP-ps or MP-ps was heated in a water bath from 30 to 90 °C for 30 min and centrifuged at 5000× *g*. The decanted hydrates were weighed, while the supernatant was transferred to a dish and dried at 65 °C to constant weight. All measurements were performed in triplicate. WHC, S and SP were calculated according to the following formulas:

WHC = (*W_h_* − *W_t_*)/*W_t_* (1)

*S* = *W_WS_*/*W_i_* (2)

SP = *W_SS_/W_i_* (1 − *S*) (3)

where *W_t_* is the total weight (g), *W_h_* is the weight of the hydrated pellet (g), *W_i_* is the initial weight (g), *W_SS_* is the weight of the decanted hydrates (g), and *W_WS_* is the weight of the dried pellet (g).

The rheological properties of *P. lobata* particles were measured using a rheometer (DHR-3, TA Instruments, New Castle, DE, USA) [24], equipped with a parallel plate geometry (diameter 40 mm, gap 1 mm). Apparent viscosity and shear stress profiles were obtained using a shear rate ramp from 0.1 to 100 s^−1^. Viscoelastic properties were determined via frequency sweep tests (0.1–100 rad/s, 1% strain).

The in vitro release profiles of puerarin from *P. lobata* particles were determined in neutral (pH 7.0) and sequential simulated gastrointestinal (pH 1.2/6.8) buffer solutions using the paddle method [25]. OP-ps or MP-ps (1.000 ± 0.001 g, *n* = 3) were incubated in neutral medium under incubation at 37.0 ± 0.5 °C with agitation at 80 rpm. Aliquots (1.00 mL) were withdrawn at predetermined time intervals (2, 5, 10, 15, 30, 45, 60, and 90 min) and immediately replaced with an equal volume of fresh pre-warmed medium. Then, OP-ps or MP-ps (1.000 ± 0.001 g, *n* = 3) were incubated in simulated gastric fluid (SGF) for 120 min and the medium was subsequently replaced with simulated intestinal fluid (SIF) for an additional 180 min. The sampling procedure was repeated at specified intervals at 2, 5, 10, 15, 30, 45, 60, 120, 125, 135, 150, 180, 210, 240, and 300 min, and the aliquot was immediately replaced with an equal volume of fresh pre-warmed corresponding medium. The puerarin concentration was quantified by high-performance liquid chromatography (HPLC; Agilent Technologies, Santa Clara, CA, USA) using an Agilent 1260 system equipped with a C8 column (CAPCELL PAK MGIII, 4.6 × 250 mm; Osaka Soda Co., Ltd., Osaka, Japan). The mobile phase consisted of methanol and water (35:65, *v*/*v*), and was delivered at a flow rate of 1.0 mL/min. The injection volume was 10 μL, the column temperature was maintained at 30 °C, and detection was performed at a wavelength of 250 nm.

### 2.4. Preparation, Optimization, and Characterization of P. lobata Particle-Based PE

A multi-factor experimental design was employed to evaluate the effects of particle type, particle concentration (1 to 5 wt%), and oil-to-water ratio (1:9, 2:8, and 3:7 *v*/*v*) on Pickering emulsion formation. Briefly, based on the preparation principles of oil-in-water emulsion systems [26], predetermined proportions of corn oil (oil phase) were mixed with deionized water (aqueous phase) containing specific amounts of OP-ps or MP-ps. The mixtures were then homogenized using a high-speed homogenizer (IKA T-25, IKA-Werke GmbH & Co. KG, Staufen, Germany) at 10,000 rpm for 10 min to construct PE. The resulting emulsions stabilized by OP-ps and MP-ps were designated as OP-p-based PE and MP-p-based PE, respectively. The storage stability of all emulsions was evaluated after 7 days at 25 °C by phase separation, in order to identify the optimal formulation for subsequent studies [27].

Capsaicin-encapsulated PEs were prepared using the obtained optimized parameters. Taking into account the functional dosage requirements of capsaicin for its biological activities [28], capsaicin was dissolved in the oil phase at specific concentrations, and homogenized with an aqueous dispersion of MP-ps at 10,000 rpm for 10 min. The resulting emulsions were designated as MP-p-PE@Cap_L_ (2 mg/mL, L), MP-p-PE@Cap_M_ (4 mg/mL, M), and MP-p-PE@Cap_H_ (6 mg/mL, H) according to capsaicin loading. As a control, OP-p/Cap was prepared identically. The physical stability of all emulsions was evaluated after 7 days of storage. Consistent with formulation optimization strategies [29], the medium-dose formulation was selected for subsequent studies. The microstructure of the MP-p-based PE@Cap_M_ and OP-p/Cap was examined using transmission electron microscopy (TEM; Talos F200S, Thermo Fisher Scientific, Waltham, MA, USA).

### 2.5. Gastrointestinal Migration Behavior of MP-p-Based PE

The in vivo gastrointestinal migration behavior and relative gastrointestinal distribution potential of the PEs were assessed after oral gavage to mice [30]. Specific-pathogen-free KM mice were randomly divided into two groups (*n* = 10 for each). The distribution and retention of Nile Red-labeled PE within the gastrointestinal tract were monitored for up to 24 h using an in vivo imaging system (IVIS). MP-p-based PE@Nile Red was prepared according to the aforementioned preparation method, with the fluorescent dye dissolved in the oil phase, while the OP-p/Nile Red suspension was prepared identically to serve as the counterpart. After oral administration of the Nile Red-labeled formulation, mice were euthanized at 2, 4, 8, and 24 h. The entire gastrointestinal tracts from stomach to colon were immediately excised and subjected to fluorescence imaging using an IVIS (IVIS Spectrum, PerkinElmer, Waltham, MA, USA).

### 2.6. In Vivo Preference Assessment of MP-p-Based PE@Cap

The potential of the MP-p-based PE to reduce oral capsaicin irritation was evaluated by a licking preference experiment [31]. Male rats were selected to avoid potential confounding effects of the estrous cycle [32]. After 24 h of water deprivation, Sprague-Dawley rats were randomly assigned to six experimental groups (*n* = 6 for each) and each group was presented with different test samples for 5 min. The samples included ultrapure water (negative control, −); capsaicin suspensions at 2, 4, and 6 mg/mL (with the 4 mg/mL suspension serving as the positive control, +); blank MP-p-based PE; and MP-p-based PE@Cap (4 mg/mL). The entire procedure was video-recorded. The cumulative licking time was analyzed via repeated-measures ANOVA to evaluate differences in acceptance.

### 2.7. In Vivo Gastrointestinal Mucosal Assessment of MP-p-Based PE@Cap

The potential of the MP-p-based PE to reduce gastrointestinal capsaicin irritation was evaluated. The capsaicin dosage was kept at 40 mg/kg body weight for all formulations containing capsaicin [28]. Following the acclimation period, KM mice were randomly divided into four groups (*n* = 6 for each), which received once-daily oral gavage of ultrapure water (negative control, −), capsaicin suspension (Cap Susp., positive control, +), blank MP-p-based PE, or MP-p-based PE@Cap. During the seven-day experiment, body weight and water intake were recorded daily to monitor general physiological status and irritation-related drinking behavior. After euthanasia, the stomach, jejunum, ileum, and colon were immediately excised, flushed with PBS to remove luminal contents, and fixed in 10% formalin for 24 h at room temperature. The fixed tissues were then dehydrated through a graded ethanol series, embedded in paraffin, sectioned at a thickness of 5 μm, and stained with H&E. Histological morphology was observed using an inverted fluorescence microscope (IX71; Olympus, Tokyo, Japan) at 200× magnification.

### 2.8. Statistical Analysis

All experiments were performed at least three times and were reported as the mean ± standard deviation (SD). Analysis of variance (ANOVA) with Tukey’s post hoc test (*p* < 0.05) was performed to compare data sets, using IBM SPSS Statistics (version 26.0, SPSS Inc., Chicago, IL, USA).

## 3. Results and Discussion

### 3.1. Microwave-Assisted Enzymolysis Provides a Simple Strategy for P. lobata Particle Modification

**Figure 1:**
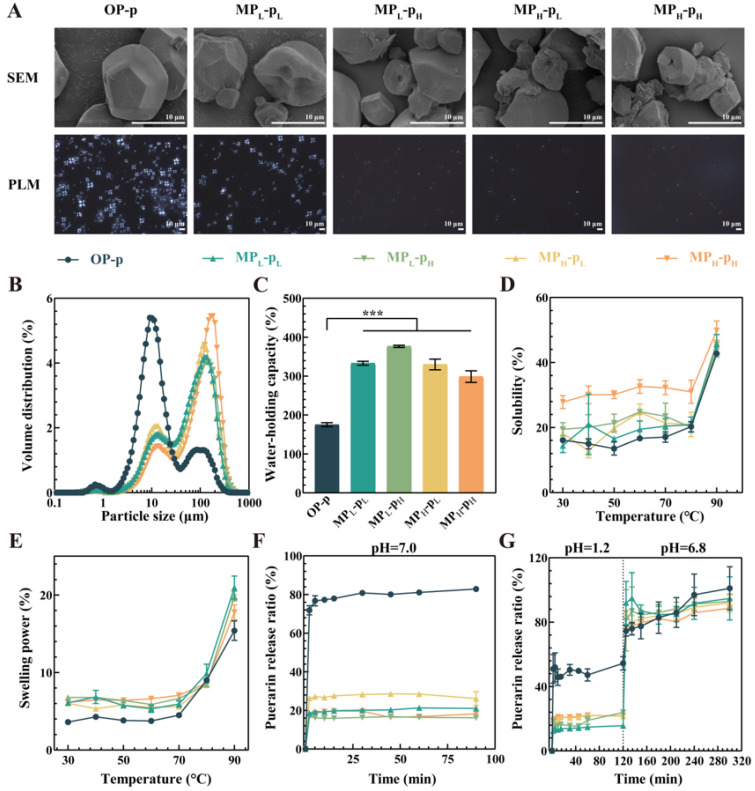
Effects of microwave-assisted enzymolysis modification on the properties of *P. lobata* particles. (**A**) SEM and PLM images of original *P. lobata* particles (OP-ps) and modified *P. lobata* particles (MP-ps); (**B**–**E**) size distribution (**B**), water-holding capacity (**C**), solubility (**D**) and swelling power (**E**) of OP-ps and MP-ps; (**F**,**G**) *in vitro* release profiles of puerarin from the particles in neutral medium (**F**) and simulated gastrointestinal fluids (**G**). Data are presented as mean ± standard deviation (*n* = 3). *** *p* < 0.001.

Firstly, the microwave-assisted enzymolysis modification altered the particle morphology and size of *P. lobata* particles. Original *P. lobata* particles (OP-ps) were composed of irregularly shaped particles, with wide size distribution ranging from 10.16 to 14.14 μm. In SEM images, massive starch granules, with polyhedral morphology and smooth surfaces, were scattered in the field of view or embedded in the fiber bundle structure (Figure 1A). Under polarized light, OP-ps exhibited obvious Maltese Cross, indicating the crystallization of starch granules. After microwave-assisted enzymolysis, cracks and pores appeared on the particles and varying degrees of agglomeration occurred. Stronger microwave energy led to a higher degree of starch granule expansion, cavitation, and fracture, while a lower water amount during microwave processing led to a higher degree of particle agglomeration. Compared with OP-ps, the D_10_, D_50_, and D_90_ values of modified *P. lobata* particles (MP-ps) were significantly increased and the polarized Maltese Cross of MP-ps was decreased (Figure 1B). Higher microwave energy during microwave processing led to larger particle sizes and weaker light birefringence. This might be due to the increased surface roughness, which increased enzyme active sites, improved the efficiency of enzymatic hydrolysis and produced more amylose chains [33]. Then, the short amylose chains resulted in the formation of RS during retrogradation [34].

Secondly, the microwave-assisted enzymolysis modification also altered the hydration properties of *P. lobata* particles (Figure 1C). The water-holding capacity of MP-ps, especially of MP_L_-p_H_ (376.39 ± 0.03%), was significantly higher than that of OP-ps (175.13 ± 0.05%). The solubility (Figure 1D) and swelling power (Figure 1E) of all the OP-ps and MP-ps in water increased with temperature, and reached the highest level at 90 °C. Among these, MP_H_-p_H_ had much higher solubility and swelling power than the others. All the OP-p and MP-p pastes (1%, wt%) were pseudoplastic (shear-thinning) fluids, exhibiting reducing apparent viscosity with increasing shear rate, and more elastic than viscous (G’ > G”) under the same angular frequency (Figures S1 and S2). The absolute viscosity of all the MP-p pastes was higher than that of OP-p pastes, suggesting that MP-ps formed a stronger gel network upon hydration. The microwave-assisted enzymolysis modification provided ample specific surface area, pathways, and hydrophilic groups for water permeation and storage. The outstanding hydration property of MP-ps gave them the potential to act as particle stabilizers in PE construction [35].

Finally, the microwave-assisted enzymolysis modification significantly changed the release behavior of puerarin from *P. lobata* particles. During in vitro incubation, the puerarin in OP-ps dissolved quickly and completely. In neutral medium, the cumulative release amount was (72 ± 2)% within 2 min (Figure 1F). In a sequential simulated gastrointestinal system, the cumulative release amount in simulated gastric fluid (SGF) was (54 ± 4)% after 120 min incubation, and increased to (86 ± 1)% following 180 min incubation in simulated intestinal fluid (SIF) (Figure 1G). In contrast, the release behavior of all MP-ps showed a significant reduction in early burst release, whether in neutral or in acidic medium. In neutral medium, the cumulative release amount rapidly reached a plateau stage of only 20%. In the sequential simulated gastrointestinal system, about 20% puerarin was released during the 120 min incubation in SGF and about 95% puerarin released during the followed SIF incubation. These results suggested that the microwave-assisted enzymolysis gave MP-ps the capacity of intestine-targeting delivery. This should be attributed to the RS in modified MP-ps, which acted as a physical barrier against acid digestion.

### 3.2. Modified P. lobata Particles Can Act as the Pickering Stabilizer for PE Construction

**Figure 2:**
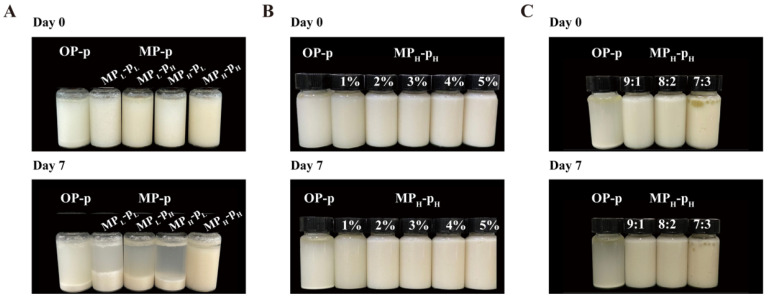
The governed optimization of PE construction. (**A**) Effect of particle modification on PE formation. (**B**,**C**) Effect of particle concentration (**B**) and water-to-oil ratio (**C**) on PE stability at a fixed particle concentration of 4 wt%.

The influences of particle modification and addition quantity, as well as water-to-oil ratio, on PE formation were systematically analyzed, taking emulsion morphology and storage stability as the evaluation indices. OP-ps were unable to form stable emulsions, as complete phase separation or oil leakage could be observed even within 1 day post-formation (Figure 2A). MP-ps were able to form PEs, but different parameters of *P. lobata* particle modification and emulsion preparation led to different storage stabilities (Figure 2B,C). After 7 days storage, the PEs based on MP_H_-p_H_ were still highly homogenous. In addition, higher particle concentration in the water phase and a higher water-to-oil ratio led to greater emulsion stability. At low particle concentrations, insufficient interfacial coverage can lead to droplet coalescence and phase separation, whereas an appropriate amount of particles can form a denser interfacial barrier. Thus, the optimal preparation parameters for *P. lobata*-based PE (MP-p-based PE) construction were 4 wt% stabilizer MP_H_-p_H_ and a 9:1 (*v*/*v*) water-to-oil ratio.

### 3.3. Capsaicin-Encapsulated PE Could Be Successfully Constructed Based on Modified P. lobata Particles

**Figure 3:**
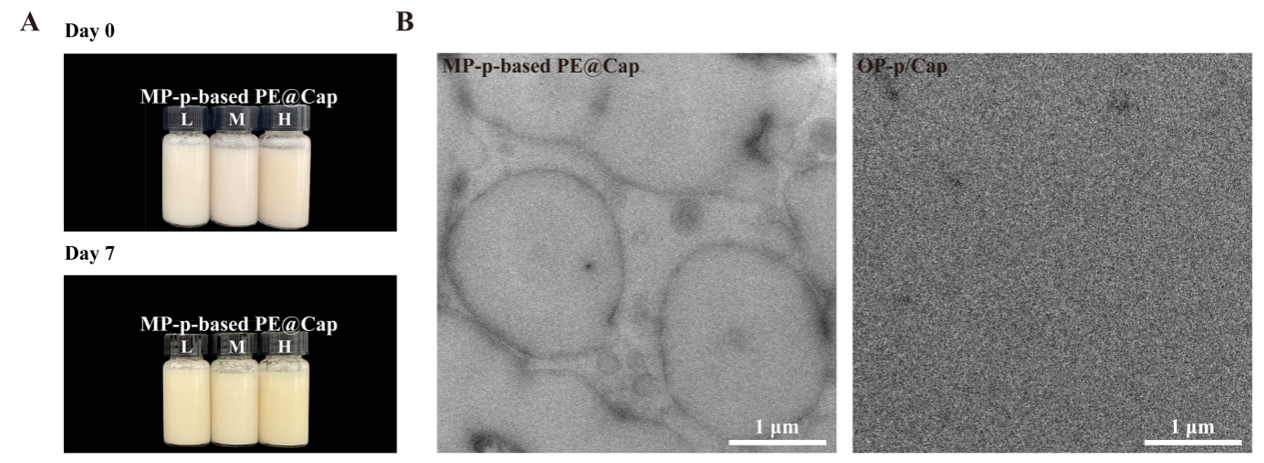
Stability and microstructure of capsaicin-encapsulated MP-p-based PE. (**A**) Macroscopic appearance of MP-p-based PE@Cap_L/M/H_ after 7-day storage at 25 °C; (**B**) TEM images of the MP-p-based PE@Cap (left) and the OP-p/Cap (right).

Capsaicin-encapsulated MP-p-based PEs (MP-p-based PE@Cap_L/M/H_) were prepared according the optimal parameters. They maintained milky-white appearances without phase separation during 7-day storage at 25 °C, demonstrating satisfactory system stability (Figure 3A). In TEM images, MP-p-based PE@Cap droplets maintained spherical core–shell architectures, with MP-ps as the solid particle stabilizer coated around the oil droplets (Figure 3B). In contrast to the MP-p-based PE, which stably encapsulated capsaicin, the sample prepared with OP-ps exhibited obvious phase separation. This suggests that modified *P. lobata* particles formed a mechanical barrier that suppresses droplet coalescence [36], serving to encapsulate lipophilic/irritant substances.

### 3.4. MP-p-Based PE Could Deliver Lipophilic Substances with Sustained and Enhanced Colonic Retention

**Figure 4:**
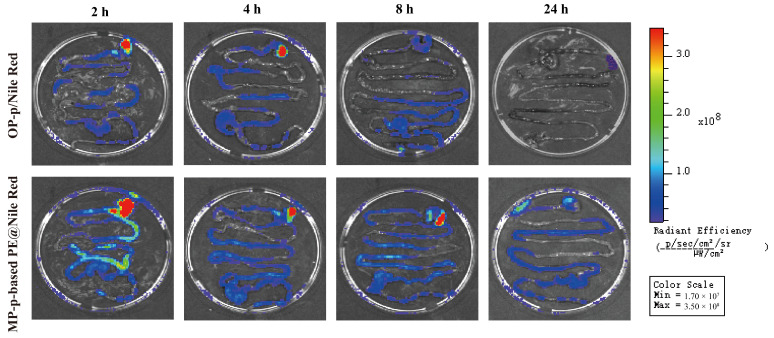
In vivo gastrointestinal tract fluorescence of OP-p/Nile Red and MP-p-based PE@ Nile Red at 2, 4, 8, and 24 h post-administration.

In order to evaluate the migration profile in the digestive tract, OP-p/Nile Red and MP-p-based PE@Nile Red were prepared and observed by an IVIS after oral gavage (Figure 4). The OP-p/Nile Red exhibited rapid gastrointestinal transit characteristics, with fluorescence signal diminution from the stomach 4 h post-administration, complete evacuation from the ileum 8 h post-administration, and clearance from the entire tract 24 h post-administration. In contrast, MP-p-based PE@Nile Red exhibited a longer residence time in the gastrointestinal tract. During the initial 2 h post-administration, the fluorescent signal was distributed homogeneously in the stomach and upper intestine; 4 h post-gavage, the fluorescence intensity in the ileum increased while the jejunal signals remained stable; eight hours post-gavage, the fluorescence signal revealed retention in the ileocolonic region; ultimately, the fluorescence signal was strong in the colon 24 h post-gavage. Crucially, the fluorescence signal remaining in colon was stronger than that of OP-p/Nile Red even at 24 h. These results indicated that MP-p-based PE@Nile Red not only exhibited a significantly prolonged intestinal retention time but also accumulated preferentially in the colon. This may be attributed to the oil droplet compartment and the MP-p interfacial layer. Moreover, the digestion resistance of MP-ps may help maintain the emulsion structure during upper gastrointestinal digestion and support the subsequent release of capsaicin in the lower gastrointestinal environment.

### 3.5. MP-p-Based PE Could Significantly Reduce the Irritation of Capsaicin

**Figure 5:**
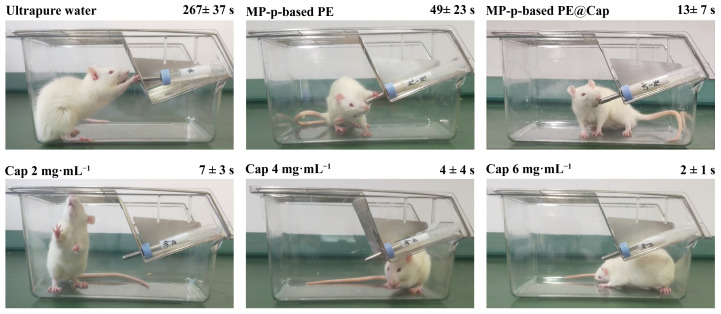
Preference behavior analysis for MP-p-based PE as an oral capsaicin delivery system. Data are expressed as mean ± SD (*n* = 6).

**Figure 6:**
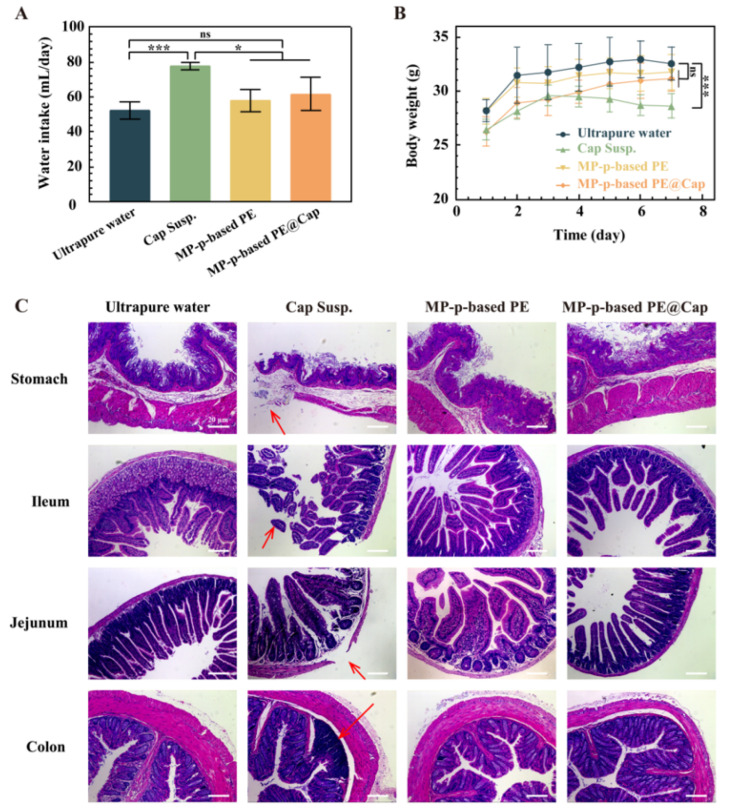
In vivo irritation assessment of MP-p-based PE@Cap. (**A**,**B**) Changes in body weight (**A**) and water intake (**B**) monitored during the 7-day oral gavage period (*n* = 6); (**C**) H&E-stained sections of the gastrointestinal tract (stomach, jejunum, ileum, and colon). Ultrapure water was used as the negative control (−), and capsaicin suspension was used as the irritation positive control (+). Data are presented as mean ± SD. *** *p* < 0.001, * *p* < 0.05, ns: not significant. Scale bar = 20 μm.

In the licking preference experiment (Figure 5), water-deprived rats showed near-total rejection of capsaicin suspensions in the 5 min observation window, with only (7 ± 3), (4 ± 4), and (2 ± 1) s licking of the 2, 4, and 6 mg/mL capsaicin suspensions, respectively. MP-p-based PE@Cap (4 mg/mL) resulted in a longer licking time—(13 ± 7) s—than the capsaicin suspension with the same concentration. The licking time of the blank PE was (49 ± 23) s, indicating the palatability of the PE construction. These results indicated that encapsulation in the MP-p-based PE partially reduced capsaicin-induced oral rejection. In the irritation experiment (Figure 6A,B), the oral gavage of capsaicin suspensions (4 mg/mL) significantly increased the water intake and reduced the body weight of KM mice. The increased water intake may reflect capsaicin-induced oral and gastrointestinal discomfort, whereas suppressed body weight gain suggests impaired physiological tolerance during repeated administration. In the MP-p-based PE@Cap group, these hyperdipsic responses were effectively ameliorated, and the mice exhibited steady body weight gain.

H&E staining analysis further revealed the ability of MP-p-based PE to reduce capsaicin irritation in the gastrointestinal tract (Figure 6C). The mice who received ultrapure water (control group, −) maintained preserved gastric mucosal folds, continuous villous structures in the jejunum/ileum/colon, and intact colonic epithelium. In contrast, the capsaicin suspension (+) caused severe vacuolation in gastric tissue, and broken villi and distorted crypts in intestinal tissue. The administration of MP-p-based PE@Cap did not influence the preserved intact gastric and intestinal structures, maintaining intact villus height and crypt morphology. The mice in the blank PE group exhibited a similar structure to that of the mice in the control group, indicating the inherent safety of the PE system. These results demonstrated that the MP-p-based PE delivery system mitigated the irritation of capsaicin.

Capsaicin serves as a health-promoting ingredient in functional food products due to its diverse biological activities. However, the intense gastric irritation of capsaicin makes it unbearable for some people, since transient receptor potential vanilloid 1 (TRPV1) is strongly expressed in the upper GI tract [37]. Flavoring agents can mask pungent taste but fail to prevent the interaction with and activation of TRPV1. Previous studies showed that the entrapment of capsaicin into microgels [38], nanoemulsions [39], and micelles [40] can mitigate the gastric irritation, but the production and application of them are usually subject to the complex preparation, high cost, and potential risk of artificial carrier materials. This study proposed a food-derived oral PE system for capsaicin delivery, using natural particles as the Pickering stabilizer. As a traditional food and medicinal material, *P. lobata* was selected to construct PE owing to its unique composition, such as high starch content, dietary fiber, and isoflavones. A simple and green modification method of starch, microwave-assisted enzymolysis, could tailor the surface wettability of *P. lobata* particles through altering starch properties. Microwave processing untangles the entanglements between the starch chains by inducing violent movement of the chains in a short time [41], creating favorable conditions for subsequent enzymatic hydrolysis [42]. Pullulanase hydrolyzes the α-1,6 linkages of amylopectin [43] and generates abundant short linear chains, leading to easier linear-chain rearrangement and recrystallization during cooling storage [44], the increase in RS proportion, and the resistance to amylase digestion in the upper gastrointestinal tract. Thus, by forming an interfacial barrier to sequester capsaicin within the oil phase, the PE system constructed with our modified *P. lobata* particles could effectively reduce its contact with upper gastrointestinal TRPV1 and enable colon-specific delivery.

Moreover, the role of our PE was moved from a passive “delivery platform” to an active “health-promoting partner”. This functional shift is attributed to the inherent active compounds (puerarin, daidzin, daidzein, and so on) of *P. lobata*, which confer beneficial effects of restoring intestinal barrier function [45], enhancing immune regulatory function [46], and modulating intestinal microbiota [47]. The PE system alleviated gastrointestinal irritation from capsaicin, as evidenced by the marked recovery in physiological parameters, including body weight and water intake. The masking effect of the PE system on capsaicin’s pungency may be limited by its own flavor. This work established a safe, low-cost and versatile platform for lipophilic/irritant substance delivery, catering to diverse delivery requirements.

## 4. Conclusions

In summary, an MP-p-based PE system was developed for the precise delivery of capsaicin. A simple and green technique of microwave-assisted enzymolysis was employed to tailor the morphology and surface properties of natural *P. lobata* particles, enabling them to act as a solid particle stabilizer for PE construction. The structural modification attenuated the rapid release of endogenous puerarin, supporting a more sustained release profile during simulated digestion. Owing to alterations in the crystalline regions of their starch granules, MP-ps exhibited enhanced resistance to upper gastrointestinal digestion and a delayed intestinal release profile. Meanwhile, the MP-p-stabilized interfacial layer facilitated capsaicin encapsulation and reduced its direct exposure in the upper gastrointestinal tract, contributing to the alleviation of capsaicin-induced oral rejection and gastrointestinal irritation. The inherent bioactive components of MP-ps, such as resistant starch, dietary fiber, and isoflavones, may provide additional nutritional functions in cooperation with capsaicin. By integrating the structural properties of natural food-derived materials with their health-promoting functions, this work provided a simple, green, and low-cost framework for oral lipophilic/irritant substance delivery systems.

## Data Availability

The original contributions presented in this study are included in the article/Supplementary Materials. Further inquiries can be directed to the corresponding author.

## Supplementary Materials

The following supporting information can be downloaded at: https://www.mdpi.com/article/10.3390/foods15152617/s1, Figure S1: Steady rheological properties of *P. lobata* particles; Figure S2: Dynamic rheological properties of *P. lobata* particles.

## Abbreviations

The following abbreviations are used in this manuscript:

Cap	Capsaicin
IVIS	In vivo imaging system
MP-p	Modified *P. lobata* particle
OP-p	Original *P. lobata* particle
PE	Pickering emulsion
RS	Resistant starch
SGF	Simulated gastric fluid
SIF	Simulated intestinal fluid
TRPV1	Transient receptor potential vanilloid 1

## References

[1] Persson M.S.M., Stocks J., Walsh D.A., Doherty M., Zhang W. (2018). The relative efficacy of topical non-steroidal anti-inflammatory drugs and capsaicin in osteoarthritis: A network meta-analysis of randomised controlled trials. Osteoarthr. Cartil..

[2] Abdulaal W.H., Asfour H.Z., Helmi N., Al Sadoun H., Eldakhakhny B., Alhakamy N.A., Alqarni H.M., Alzahrani S.A.M., El-Moselhy M.A., Sharkawi S.S. (2024). Capsaicin ameliorate pulmonary fibrosis via antioxidant Nrf-2/PPAR-γ pathway activation and inflammatory TGF-β1/NF-κB/COX II pathway inhibition. Front. Pharmacol..

[3] Brown K.C., Modi K.J., Light R.S., Cox A.J., Long T.E., Gadepalli R.S., Rimoldi J.M., Miles S.L., Rankin G., Valentovic M. (2023). Anticancer Activity of Region B Capsaicin Analogs. J. Med. Chem..

[4] Thornton T., Mills D., Bliss E. (2023). Capsaicin: A Potential Treatment to Improve Cerebrovascular Function and Cognition in Obesity and Ageing. Nutrients.

[5] Nava-Ochoa A.E., Antunes-Ricardo M., Guajardo-Flores D. (2021). Nano-sized carriers for capsaicinoids with topic analgesic and anti-inflammatory effects. J. Biotechnol..

[6] Cheng P., Wu J., Zong G., Wang F., Deng R., Tao R., Qian C., Shan Y., Wang A., Zhao Y. (2023). Capsaicin shapes gut microbiota and pre-metastatic niche to facilitate cancer metastasis to liver. Pharmacol. Res..

[7] Wang N., Zhou X., Zhang T., Jian W., Sun Z., Qi P., Feng Y., Liu H., Liu L., Yang S. (2025). Capsaicin from chili peppers and its analogues and their valued applications: An updated literature review. Food Res. Int..

[8] Wang X., Tian N., He L., Yuan Z., Han L. (2025). Emerging Applications of Pickering Emulsions in Pharmaceutical Formulations: A Comprehensive Review. Int. J. Nanomed..

[9] Rayees R., Gani A., Noor N., Ayoub A., Ashraf Z.U. (2024). General approaches to biopolymer-based Pickering emulsions. Int. J. Biol. Macromol..

[10] Pokorski P., Strojny-Cieślak B., Domian E., Załęcki M., Grygier A., Pruchniewski M., Zakrzewska A., Aktaş H., Aljewicz M., Kmiecik D. (2025). Fabrication and characterization of novel β-sitosterol-loaded O/W Pickering emulsions stabilized by edible insects protein/chitosan complex coacervates: Retention and stability evaluation. Carbohydr. Polym..

[11] Zhang Y., Tian W., Deng Y., Cao Y., Xiao J. (2025). Regulation of whey protein microparticle adsorption and interfacial film formation by polysaccharides for enhanced Pickering emulsion stability. Food Hydrocoll..

[12] Liu W., Liu J., Salt L.J., Ridout M.J., Han J., Wilde P.J. (2019). Structural stability of liposome-stabilized oil-in-water pickering emulsions and their fate during in vitro digestion. Food Funct..

[13] Feng H., Li T., Zhou L., Chen L., Lyu Q., Liu G., Wang X., Chen X. (2024). Potato starch/naringenin complexes for high-stability Pickering emulsions: Structure, properties, and emulsion stabilization mechanism. Int. J. Biol. Macromol..

[14] Guida C., Aguiar A.C., Magalhães A.E.R., Soares M.G., Cunha R.L. (2024). Impact of ultrasound process on cassava starch nanoparticles and Pickering emulsions stability. Food Res. Int..

[15] Song X., Gong H., Zhu W., Wang J., Zhai Y., Lin S. (2022). Pickering emulsion stabilized by composite-modified waxy corn starch particles. Int. J. Biol. Macromol..

[16] Zhang Y., He H., Feng S., Bi J., Huang X., Xiong J., Chen L., Chen H., Li X., Chen L. (2025). Effect of grapefruit peel pectin on the structure, pasting characteristics, and in vitro digestibility of starch under different moisture content and temperature. Int. J. Biol. Macromol..

[17] Muhammad Z., Ramzan R., Zhang R., Zhao D., Khalid N., Deng M., Dong L., Aziz M., Batool R., Zhang M. (2022). Enhanced Bioaccessibility of Microencapsulated Puerarin Delivered by Pickering Emulsions Stabilized with OSA-Modified Hydrolyzed Pueraria montana Starch: In Vitro Release, Storage Stability, and Physicochemical Properties. Foods.

[18] Zhang L., Chen D.L., Wang X.F., Qian J.Y., He X.D. (2022). Enzymatically modified quinoa starch-based Pickering emulsion: Effect of enzymolysis and emulsifying conditions. Int. J. Biol. Macromol..

[19] Wu Q., Chen K., Ma L., Man S. (2025). Pueraria: A Comprehensive Review of Chemical Composition, Health Benefits, and Current Applications. Compr. Rev. Food Sci. Food Saf..

[20] Meng F., Guo B., Ma Y.Q., Li K.W., Niu F.J. (2022). Puerarin: A review of its mechanisms of action and clinical studies in ophthalmology. Phytomedicine.

[21] Hu H., Chen B., Zhang H., Yin W., McClements D.J., Ji H., Jin Z., Qiu C. (2025). Recrystallization of optimized debranched starch: Effects of concentration on resistant starch formation and structural properties. Food Chem..

[22] Bai C., Zhang S., Huang L., Wang H., Wang W., Ye Q. (2015). Starch-based hydrogel loading with carbendazim for controlled-release and water absorption. Carbohydr. Polym..

[23] Ge X., Shen H., Su C., Zhang B., Zhang Q., Jiang H., Li W. (2021). The improving effects of cold plasma on multi-scale structure, physicochemical and digestive properties of dry heated red adzuki bean starch. Food Chem..

[24] Katepalli H., John V.T., Tripathi A., Bose A. (2017). Microstructure and rheology of particle stabilized emulsions: Effects of particle shape and inter-particle interactions. J. Colloid Interface Sci..

[25] Yang J., Sun Y., Dong X., Li M., Qin Y., Dai L., Sun Q. (2025). Interaction of starch nanoparticles with digestive enzymes and its effect on the release of polyphenols in simulated gastrointestinal fluids. Food Chem..

[26] Nimaming N., Sadeghpour A., Murray B.S., Sarkar A. (2024). Pickering oil-in-water emulsions stabilized by hybrid plant protein-flavonoid conjugate particles. Food Hydrocoll..

[27] Dong H., Ding Q., Jiang Y., Li X., Han W. (2021). Pickering emulsions stabilized by spherical cellulose nanocrystals. Carbohydr. Polym..

[28] Tang T., Song J., Wang H., Zhang Y., Xin J., Suo H. (2021). Qingke β-glucan synergizes with a β-glucan-utilizing Lactobacillus strain to relieve capsaicin-induced gastrointestinal injury in mice. Int. J. Biol. Macromol..

[29] Xiao J., Li C., Huang Q. (2015). Kafirin Nanoparticle-Stabilized Pickering Emulsions as Oral Delivery Vehicles: Physicochemical Stability and in vitro Digestion Profile. J. Agric. Food Chem..

[30] Zhang J., Yin Y.J., Wang X.W., Lu W.Q., Chen Z.Y., Yu C.H., Ren K.F., Xu C.F. (2025). Adhesive polyelectrolyte coating through UV-triggered polymerization on PLGA particles for enhanced drug delivery to inflammatory intestinal mucosa. J. Nanobiotechnol..

[31] He L.W., Zeng L., Tian N., Li Y., He T., Tan D.M., Zhang Q., Tan Y. (2020). Optimization of food deprivation and sucrose preference test in SD rat model undergoing chronic unpredictable mild stress. Anim. Models Exp. Med..

[32] Moloney R.D., Sajjad J., Foley T., Felice V.D., Dinan T.G., Cryan J.F., O’Mahony S.M. (2016). Estrous cycle influences excitatory amino acid transport and visceral pain sensitivity in the rat: Effects of early-life stress. Biol. Sex Differ..

[33] Li H.T., Zhang W., Bao Y., Dhital S. (2024). Enhancing enzymatic resistance of starch through strategic application of food physical processing technologies. Crit. Rev. Food Sci. Nutr..

[34] Wang Z., Wang S., Xu Q., Kong Q., Li F., Lu L., Xu Y., Wei Y. (2023). Synthesis and Functions of Resistant Starch. Adv. Nutr..

[35] Liu C., Ma R., Shen W., Tian Y. (2025). Unraveling the impact of starch granule-associated proteins on the emulsifying ability of quinoa starch granules at multiple scales. Food Chem..

[36] Wu L., Liang Y., Lu N., Liu B. (2025). Construction, characterization, and application of immature peach powder-stabilized Pickering emulsion gel. Front. Nutr..

[37] Yang F., Zheng J. (2017). Understand spiciness: Mechanism of TRPV1 channel activation by capsaicin. Protein Cell.

[38] Yuan Y., Liu Y., He Y., Zhang B., Zhao L., Tian S., Wang Q., Chen S., Li Z., Liang S. (2022). Intestinal-targeted nanotubes-in-microgels composite carriers for capsaicin delivery and their effect for alleviation of Salmonella induced enteritis. Biomaterials.

[39] Choi A.Y., Kim C.T., Park H.Y., Kim H.O., Lee N.R., Lee K.E., Gwak H.S. (2013). Pharmacokinetic characteristics of capsaicin-loaded nanoemulsions fabricated with alginate and chitosan. J. Agric. Food Chem..

[40] Yi M., Tang X., Liang S., He R., Huang T., Lin Q., Zhang R. (2024). Effect of microwave alone and microwave-assisted modification on the physicochemical properties of starch and its application in food. Food Chem..

[41] Zhao Y., Tu D., Wang D., Xu J., Zhuang W., Wu F., Tian Y. (2024). Structural and property changes of starch derivatives under microwave field: A review. Int. J. Biol. Macromol..

[42] Guo X., Xu J., Dai X., Kong H., Li D., Li C., Ban X., Gu Z., Li Z. (2026). Effects of pre-gelatinization methods on structure and physicochemical properties of kudzu starch. Int. J. Biol. Macromol..

[43] Xie T., Zhou L., Han L., You C., Liu Z., Cui W., Cheng Z., Guo J., Zhou Z. (2024). Engineering hyperthermophilic pullulanase to efficiently utilize corn starch for production of maltooligosaccharides and glucose. Food Chem..

[44] Zhu F., Liu P. (2020). Starch gelatinization, retrogradation, and enzyme susceptibility of retrograded starch: Effect of amylopectin internal molecular structure. Food Chem..

[45] Cai G., Wu C., Mao N., Song Z., Yu L., Zhu T., Peng S., Yang Y., Liu Z., Wang D. (2022). Isolation, purification and characterization of Pueraria lobata polysaccharide and its effects on intestinal function in cyclophosphamide-treated mice. Int. J. Biol. Macromol..

[46] Li Y., Song Z., Ding J., Zhou Y., Huang T., Qian Q., Yu M., Chen W., Liu J., Lu L. (2026). Puerarin Targets MIC19 to Suppress Mitochondrial Metabolism of Tumor-Infiltrating Tregs and Enhance Anti-tumor Immunity. Adv. Sci..

[47] Xu X., Guo Y., Chen S., Ma W., Xu X., Hu S., Jin L., Sun J., Mao J., Shen C. (2022). The Positive Influence of Polyphenols Extracted From Pueraria lobata Root on the Gut Microbiota and Its Antioxidant Capability. Front. Nutr..

