# Supplementary material for: Pickering Emulsion Stabilized by Pueraria lobata-Based Stabilizer: From a Passive Carrier to an Active Partner for Oral Capsaicin Delivery
Source: Foods. 2026 Jul 26;15(15):2617. doi: 10.3390/foods15152617 (PMC13465731; doi:10.3390/foods15152617)
Supplement: Supplementary file 1 [file foods-15-02617-s001.zip › foods-4379108-supplementary.pdf]

Pickering Emulsion Stabilized by *Pueraria lobata*-Based Stabilizer: From a Passive Carrier to an Active Partner for Oral Capsaicin Delivery

Qiongliu Yu [1], Yikang Ding [1], Hanyu Wu [1], Min Luo [1], Qunying Zhang [1], Guiming Yan [2] and Ye Yang [1,2,3,4,*]

[1] School of Pharmacy, Anhui University of Chinese Medicine, Hefei 230012, China; qunyingzhang701025@ahtcm.edu.cn (Q.Z.)
[2] School of Nursing, Anhui University of Chinese Medicine, Hefei 230012, China; wuweizi@ahtcm.edu.cn
[3] Anhui Provincial Key Laboratory of Research & Development of Chinese Medicine, Hefei 230012, China
[4] Anhui Provincial Key Laboratory of Pharmaceutical Preparation Technology and Application, Hefei 230012, China
* Correspondence: y.yang@ahtcm.edu.cn

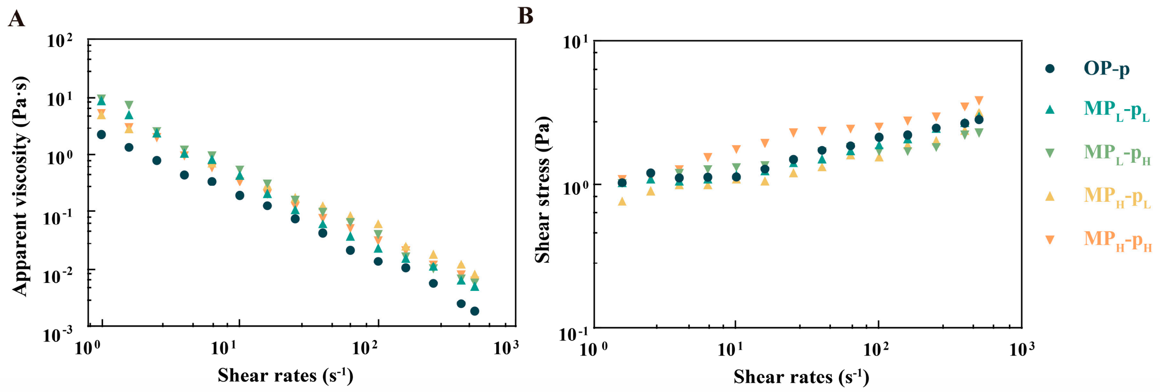

Figure S1. Steady rheological properties of *P. lobata* particles. (A) Apparent viscosity and (B) Shear stress as a function of shear rate.

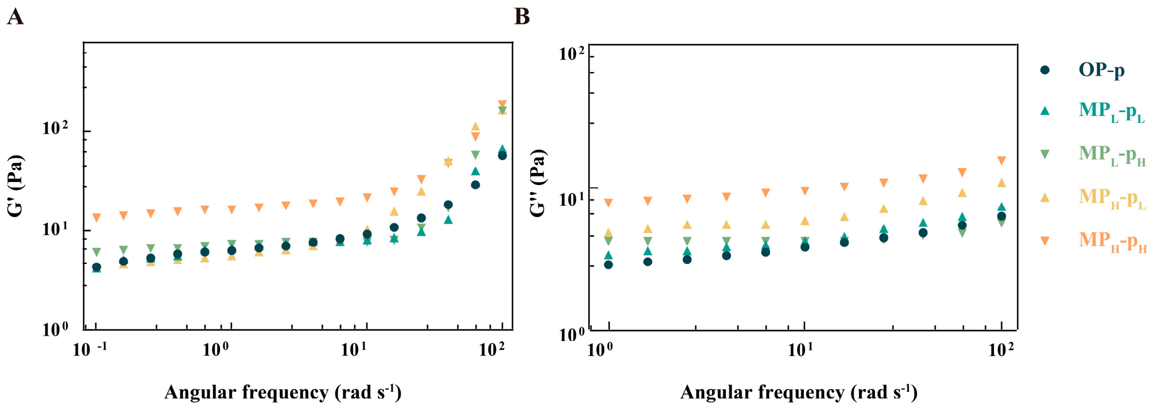

Figure S2. Dynamic rheological properties of *P. lobata* particles. (A) Storage modulus (G‘) and (B) loss modulus (G”) as functions of angular frequency.
